# Transferrin: From Inorganic Biochemistry to Medicine

**DOI:** 10.1155/MBD.1994.161

**Published:** 1994

**Authors:** Luigi Messori, Felix Kratz

**Affiliations:** 1 Department of Chemistry University of Florence Via G. Capponi 7 Florence I - 50121 Italy

## Abstract

Transferrin is one of the key proteins of iron metabolism in mammalians. It is responsible for the transfer of the essential iron(III) ions through the biological fluids from absorption to storage and utilization sites. Moreover, transferrin is involved in the metabolism of other metal ions that are either trace or toxic elements. In recent years the crystal structure of transferrin has been solved at high resolution. This has allowed an extensive reinterpretation of the many spectroscopic studies carried out on this protein in the last decade as well as the elucidation of some interesting structure-function relationships. We review here recent progresses in transferrin biochemistry, particular focus being given to those aspects that are relevant from a medical point of view.


### TRANSFERRIN: FROM INORGANIC BIOCHEMISTRY TO MEDICINE

Luigi Messori* and Felix Kratz

Department of Chemistry, University of Florence, Via G. Capponi 7, I - 50121 Florence, Italy

**Abstract**
Transferrin is one of the key proteins of iron metabolism in mammalians. It is responsible for the transfer of the essential iron(III) ions through the biological fluids from absorption to storage and utilization sites. Moreover, transferrin is involved in the metabolism of other metal ions that are either trace or toxic elements. In recent years the crystal structure of transferrin has been solved at high resolution. This has allowed an extensive reinterpretation of the many spectroscopic studies carried out on this protein in the last decade as well as the elucidation of some interesting structure-function relationships. We review here recent progresses in transferrin biochemistry, particular focus being given to those aspects that are relevant from a medical point of view.

### 1. Inorganic Biochemistry of Transferrins.

Transferrins constitute an homologous family of monomeric glycoproteins whose primary members are serum transferrin, lactoferrin and ovotransferrin [1]. Transferrins are bilobal proteins comprising about 700 amino acids, with a molecular weight of ca. 78 kD. They bind two iron(III) equivalents per molecule at two distant but very similar and apparently non-interacting sites. Metal binding is strongly promoted by the concomitant binding of an anion, the so called synergistic anion, which is carbonate under physiological conditions.

*a) X-ray studies.*

Protein crystallographic studies have shown that transferrins consist of two homologous lobes each of which comprises two inequivalent domains with the iron binding sites located within an interdomain cleft [2,3]. Such arrangement allows a large molecular flexibility and turns out to be a critical factor for the function of transferrins (Figure 1). The structure of the two virtually identical metal binding sites has been determined in detail. Iron is octahedrally coordinated by four protein donors (namely two Tyr phenolates, one His imidazole and one Asp carboxylate)

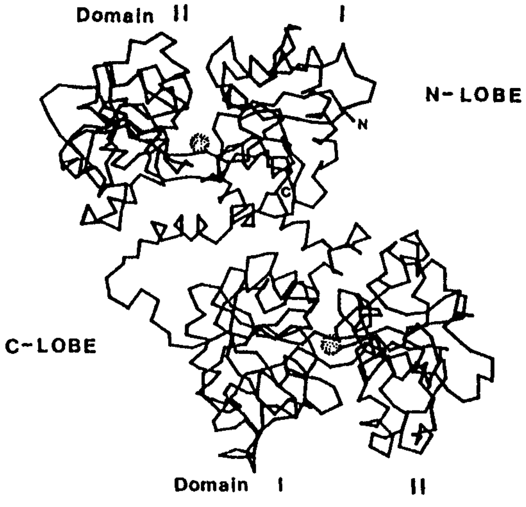

Figure 1. General structure of the protein (from ref.3 with permission).

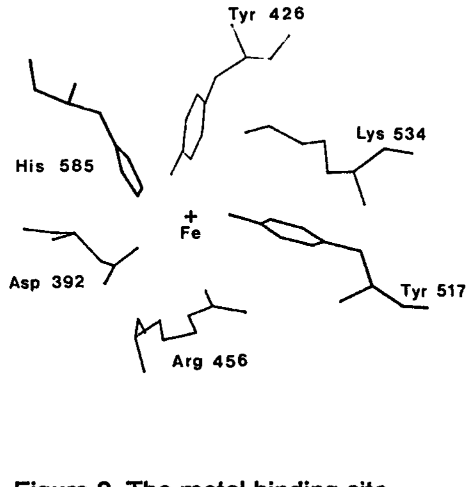

Figure 2. The metal binding site (from ref.3 with permission).

and by the bidentate carbonate anion. The carbonate anion fits into a pocket between the iron atom and two positively charged protein groups, an arginine side chain and a helix N-terminus. The structure of the metal binding sites is shown in Figure 2.

Inspection of the metal binding site structure provides an insight into the probable mechanism of metal uptake. It has been hypothesized that the protein first binds the synergistic anion and the metal at domain I, then domain II closure occurs and the coordination shell of the iron(III) ions is completed through the carboxylate group of Asp [4]. The reverse might hold in case of metal removal.

*b) The metal binding properties*

Apart from iron(III) apotransferrin can bind several other metal ions, which differ in charge and in size, with relatively high affinity. Although preferential binding of tripositive metal ions has been demonstrated -i.e. Al(III), Ga(III), In(III), Tl(III), all the lanthanides, Mn(III), Co(III), V(III), Cr(III) and Ru(III)- the protein can also bind bipositive -i.e. Ni(II), Fe(II), Co(II), Cu(II), Cd(II), Mn(II), and Zn(II)- and tetrapositive ions -i.e. Th(IV), Hf(IV) and Pu(IV). Binding invariantly occurs at the iron binding sites as shown by a number of spectroscopic studies. The ability of the protein to accomodate so many cations different in charge and size at the same binding site suggests that the metal binding site itself is very flexible. Indeed, the X-ray results have shown that the metal binding site is not preformed, but builds up around the metal cation through domain closure. This fact reasonably accounts for its flexibility. The affinity constants of human serum transferrin for several metal ions have been carefully measured by Harris through competition studies using UV difference spectroscopy; the following series of affinity constants has been found: $Fe^{3+} > Ga^{3+} >> Al^{3+} >> Zn^{2+} > Cd^{2+}$[5,6].

Another issue of major concern is the conformational equivalence among the different metal derivatives of transferrin in terms of molecular recognition of metal loaded transferrins by the specific cell receptor. It has been demonstrated that metal binding is accompanied by a large conformational change of the protein from an open to a closed conformation [7]. Small angle X-ray scattering studies suggest that the overall conformation of the protein depends on the nature of the metal; this might affect the molecular recognition properties of the different metallotransferrins by the cell receptor. In particular, it has been shown that In(III) and Cu(II) induce the same domain closure as Fe(III) but Al(III) causes a conformational change of somewhat smaller magnitude; Hf(IV) does not induce any major conformational change [8]. These findings contain important implications for the fate of the different metals in the body after binding to transferrin.

*c) The metal release reaction*

The release of iron from transferrin has been investigated in detail. There is now substantial agreement that iron release from transferrin is at least a biphasic process with two observable rate constants that probably represent release from the two distinguishable sites [1]. The more facile releasing site is the N-terminal site. Iron release from transferrin is sensitive to the ionic environment of transferrin and to the chemical nature of the iron accepting ligand. High concentrations of salt stabilize binding at the N-terminal site while facilitating release from the C-terminal site so that the relative labilities of the two sites are reversed.

The overall mechanism of iron release is as yet unclear. Two alternative pathways, perhaps complementary, have been proposed. In the first of these the iron accepting ligand forms a quaternary complex with transferrin; then the iron ion, bound to the ligand, dissociates from the protein. Conversely, the pathway proposed by Cowart et al. implies a conformational change in transferrin from a closed to an open structure in which iron dissociation occurs [9]. Recently Aisen et al. have postulated an active role of the transferrin receptor in the metal release mechanism [10]. The debate on the mechanism of iron release from transferrin has been recently surveyed by Raymond [11]. Large interest has been devoted to the nature of the ligands capable of removing iron from transferrin owing to their potential medical applications. The most effective and popular chelators are those containing catecholate, hydroxamate or phosphonate groups whereas hydroxipyridonates represent a relatively new class of iron scavenging compounds. The possible use of such ligands in iron chelation therapy [12] and in strategies of heavy metal decontamination [13] has been extensively considered.

### *d) Molecular biology studies of transferrins*

One of the most significant developments in transferrin research has been the cloning and the high level expression of several transferrins, mutants and fragments [14,15]. Interestingly, it has been shown that the half molecules of serum transferrin and lactoferrin bind iron with spectroscopic properties similar to those of the native molecules, supporting the view that the actual protein is the result of the fusion of two units of an ancestral protein. Site directed mutagenesis experiments are now being performed that have the potential to elucidate the structural and functional role of single amino acid residues in the active site of the protein. Mutagenesis experiments have so far focused primarily on the metal and anion binding residues, notably Arg 124, which forms a hydrogen bond to the carbonate anion and to the iron ligand Asp 63. Mutations of the Asp ligand cause marked spectral changes and reduce stability of iron binding. Surprisingly, mutations of Lys 206 and His 207 also induce marked spectral variations. However, the structural and functional consequences of these and other mutations have yet to be established.

### *e) The interaction of transferrin with its specific cell receptor*

Iron uptake by cells is largely mediated by the specific receptor for transferrin located on the cell surface. It is generally believed that diferric transferrin binds to its receptor and is then rapidly internalized in nonlysosomal vesicles. The acidic environment of these vesicles would cause iron to dissociate from the protein while apotransferrin remains bound to its receptor. Afterwards, the complex of transferrin and the receptor is rapidly returned to the cell surface where apotransferrin rapidly dissociates from the receptor at neutral pH [16]. The transferrin cycle is represented in Figure 3.

The molecular aspects of the transferrin receptor (TfR) have started to be clarified [17]. The TfR are disulfide linked dimers each of molecular mass 90-95 kDa corresponding to about 760 amino acid residues. The receptor is anchored in the plasma membrane by a

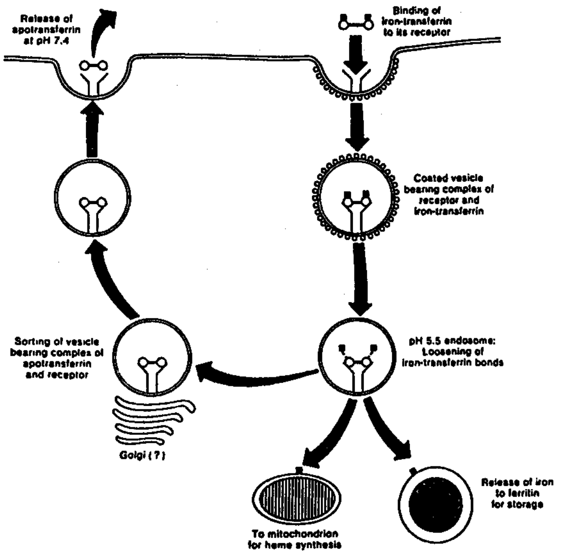

Figure 3. The transferrin cycle (from ref.1 with permission).

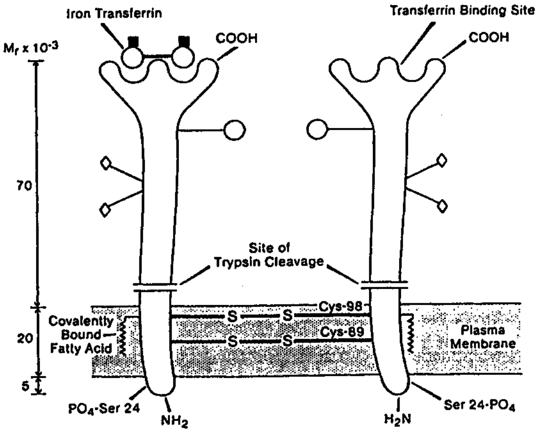

Figure 4. The transferrin receptor. (from ref.1 with permission).

transmembrane helix with an internal N-terminal domain of 61 residues and an external domain of 671 amino acid residues. Some aspects of TfR physiology and molecular biology have been elucidated. The distribution of the transferrin receptor in different cell types, the modulation of its expression and its molecular recognition properties appear to be critical factors with respect to the physiology of iron metabolism and to the use of transferrin and its derivatives in medicine.

### 2. Transferrin and Medicine.

Transferrin plays a central role in iron metabolism and therefore represents an obvious target for therapeutic strategies of iron overload. Many research efforts have been devoted to develop ligands capable of removing iron from transferrin for clinical use in iron chelation therapy. Furthermore, the ability of transferrin to bind metals other than iron has additional medical diagnostic and therapeutic implications. For example, among the non-physiological metals capable of binding transferrins, gallium(III), and indium(III) are exploited for medical use (tracer and imaging studies) whereas others like aluminum(III) and the actinides are of toxicological interest. Another aspect of transferrin chemistry, of potential medical interest, is drug targeting of cytotoxic compounds. We survey below all these aspects.

### *a) Iron metabolism and its disorders: iron chelation therapy.*

In a healthy man the total body iron is maintained at a constant level of about 4-5g by balancing the daily absorption with an average daily loss of a similar amount. Serious toxic effects can arise as a consequence of excess body iron, a rather frequent situation known as iron overload. Iron overload may occur as a consequence of thalassemia or other genetic disorders like idiopathic hemochromatosis. Treatment of iron overload is a difficult medical task and requires long-term therapy with iron chelating agents. Recent work suggests future extension of iron chelation therapy to other important areas of medical concern such as the prevention of tissue damage following myocardial infarction and the control of some bacterial infections [18].

To revert iron overload selective iron chelators must be used to sequester iron. For the past 20 years desferrioxamine B (Desferal) has been the only clinically useful drug. Desferrioxamine is a fungal siderophore and has an extremely high affinity for iron(III). The molecule is quite selective for iron possessing much lower affinities for copper, zinc, calcium and magnesium.

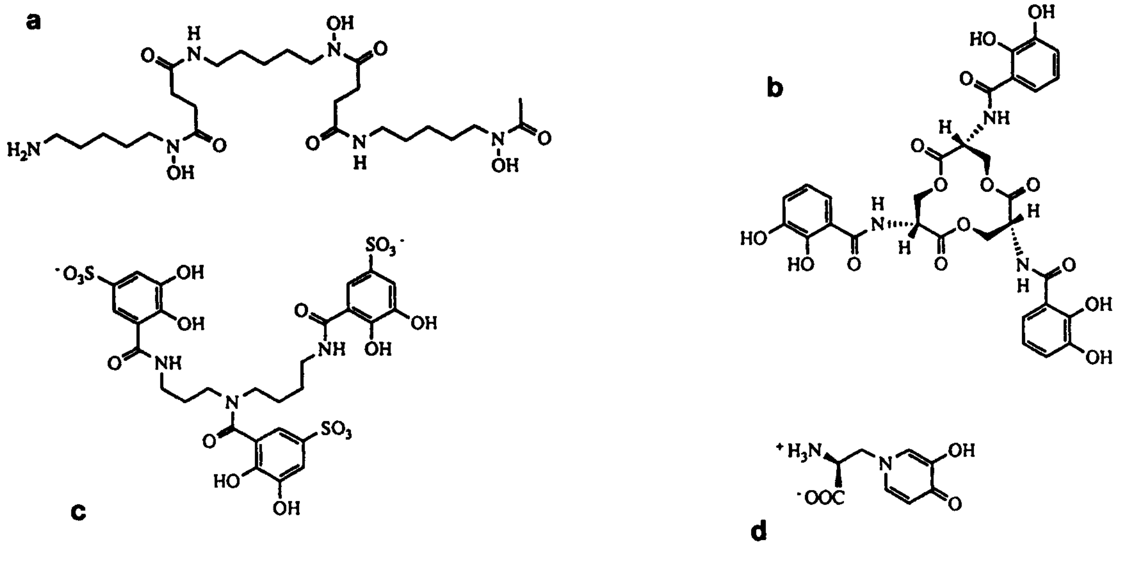

Figure 5. Structure of some iron chelators: *a*. desferrioxamine B; *b*. enterobactin; *c*. 3,4 LICAMS; *d*. L-mimosine, a hydroxypyridone ligand (from ref. 11 with permission).

These facts render the molecule particularly attractive for clinical use. However, desferrioxamine has the disadvantage of being orally inactive and only causes sufficient iron excretion when given either subcutaneously or intravenously over 8-12 h periods up to six times per week. Consequently, orally active chelators with the potential of replacing desferrioxamine in the clinic are actively sought after. Among these hydroxamate, tricatecholate and, in the last period, hydroxypyridinone ligands appear to possess rather favorable properties and are being tested in clinical trials [19].

*b) Transport of radiopharmaceuticals: gallium-67 and indium-111*

The medical literature is rich of reports on the use of gallium 67 and indium 111 to detect many types of malignancies in humans [20]. Some examples are lung cancer, Hodgkin and non Hodgkin lymphomas, malignant melanoma, breast carcinoma and Ewing sarcoma. It is now largely accepted that gallium-67 is superior to indium-111 in cancer diagnosis. When gallium-67 and indium-111 are administered parenterally in a readily dissociable form, they bind quickly to plasma transferrin. Further biodistribution processes are then mainly governed by transferrin even if not in the same way as for iron(III) ions. On the other hand, if the two radionuclides are administered in forms that do not significantly dissociate, scarce binding to transferrin occurs so that gallium-67 and indium-111 follow the biodistribution characteristics of the ligand to which they are associated.Apart from their being useful radiodiagnostic agents gallium and indium, in their stable isotopic forms, have been used, bound to transferrin, as experimental drugs to inhibit cell proliferation (see later).

*c) Heavy metal contamination and strategies for detoxification*

Heavy metals are hazardous for humans. This raises the medical problem of decontamination following acute or chronic poisoning. Since several toxic metals bind transferrin, the protein represents an obvious target for decontamination strategies. Particular interest has been devoted to the cases of accidental contamination with the extremely toxic plutonium, where a very rapid therapeutic intervention is required. Also aluminum poisoning has attracted scientific interest since the latter element appears to be correlated to the appearance of the Alzheimer disease.

*Plutonium:* Because of the extensive manipulations involving plutonium in the nuclear weapons industry accidents involving this element occur with a certain frequency. The biological transport and storage of plutonium(IV) is similar to that of iron(III) and this characteristic provides the rationale for the most recent developments in decontamination strategies. It was shown that EDTA type compounds, of which DTPA is the most effective member, are capable of significantly enhancing the excretion of Pu-239 [13,21]. The many similarities between Pu(IV) and Fe(III) stimulated the search for chelating agents with donor groups similar to those of the naturally occurring iron binding compounds, the so called siderophores, synthetized by various microrganisms. Indeed, desferrioxamine can mobilize Pu-239 from most organs with the exception of the kidney [13]. Among the other tested ligands LICAMS are the most effective ones [13,22]. It has been reported that the removal of an injected dose of Pu-239 by these chelating agents was approximately the same as found for DTPA, but the new ligands have the advantage of an even lower reactivity at physiological pH with essential divalent metal ions and the ability to remove some of the Pu(IV) already deposited in the bone. This development is a striking demonstration of the design of a new selective chelating agent for a toxic metal on the basis of an effective understanding of its coordination properties.

*Aluminum:* Although aluminum has no known biological function, a toxic amount of aluminum can cause dialysis encephalopathy in humans and is associated with other degenerative disorders such as senile dementia of the Alzheimer type. In vitro the binding of aluminum(III) to transferrin has been probed by a number of spectroscopic techniques [23,24]. Plasma speciation studies point to transferrin as the major carrier protein of aluminum(III). Recent evidence suggests that Tf from people affected with Alzheimer disease may be deficient in its ability to complex and remove aluminum contributing to the deposition of high levels of this metal in the brain [25]. In other words the aluminum toxicity would greatly increase when the binding capacity of transferrin is saturated; low molecular weight aluminum(III) complexes would form that are capable of crossing the blood-brain barrier. These findings led to the belief that the use of aluminum chelators might well provide a therapeutic rationale for the treatment of aluminum toxicity. Both apotransferrin and desferrioxamine B are being tested for the treatment of aluminum toxicity.

*d) Drug targeting*

In light of the presence of high amounts of transferrin receptors on human cancer cells, Faulk and colleagues proposed that these receptors could be utilized as targets to bind drug conjugates of transferrin [26]. A similar proposal was that transferrin receptors could be used as a target to bind drug conjugates of antibodies to transferrin receptors. The concept of drug targeting with antibody has been actively pursued with the use of antibodies conjugated with doxorubicin, methotrexate, ricin A chain and other chemotherapeutic agents; however, antibodies often cause serious problems such as severe anaphylactic reactions. These side effects caused by antibodies are reduced if the delivery system is transferrin which is the natural ligand for the transferrin receptors. Faulk has shown that transferrin conjugates of the anticancer drug adriamycin selectively target to tumor cells and the targeted cells are thus killed [27]. These conjugates have been used in the treatment of patients with acute leukemia and the results have shown diminished numbers of leukemic cells in peripheral blood, no increase of leukemic cells in bone marrow biopsies and no anaphylactic reaction in the patients. Other transferrin conjugates, like those with toxins [28] and those with some ruthenium(III) complexes [29], have been prepared and are being investigated in cell proliferation inhibition studies.

Another somewhat different example of drug targeting is represented by gallium transferrin [30]. It has been reported that gallium significantly inhibits cell growth, this inhibitory effect being potentiated by the addition of transferrin. Chitambar et al. have conducted interesting studies of the effects of gallium transferrin on cell proliferation. Initially they found that after exposure to gallium transferrin HL60 cells stop proliferating while exhibiting an increased number of transferrin receptors [31]. Subsequent studies indicated that gallium transferrin impairs intracellular release of iron from transferrin by interfering with processes responsible for intracellular acidification [32]. Finally, Chitambar et al. have shown that gallium transferrin inhibits DNA synthesis through action on the iron containing M2 subunit of ribonucleotide reductase [33]. In view of these findings, we believe that gallium transferrin deserves careful clinical evaluation for the treatment of malignancies in which tumor cells display significant numbers of transferrin receptors. For example high grade lymphomas are potential candidates to treatment with gallium transferrin.

### 3. Concluding Remarks

The knowledge of transferrin has increased in recent years thanks to the determination of its X-ray structure and to a host of spectroscopic and kinetic studies. Studies on site specific mutants obtained through recombinant techniques have now the potential to elucidate the role of individual residues in the thermodynamics and kinetics of the metal binding/release reactions. This will help designing new ligands capable of scavenging metals from transferrin with high selectivity and efficiency. Moreover, we have described the expanding role of transferrin in medicine. Transferrin indeed is involved in several processes of large biomedical concern such as treatment of iron overload, detoxification of heavy metal poisoning, delivery of radiopharmaceuticals, drug targeting, treatment of the Alzheimer disease, control of cell proliferation, etc. We feel that a deeper knowledge of the bioinorganic chemistry of transferrin will contribute significantly to these medical developments.

***Acknowledgment***

Stimulating discussions with Prof. E.N. Baker are gratefully acknowledged.

### 4. References.

1. Harris, D.C. and Aisen, P. (1989) in *Iron Carriers and Iron Proteins* (Loehr, T.M., ed.) pp. 239-351, VCH Publishers Inc. New York; *ibidem* Aisen, P. pp. 353-371.
2. Anderson, B.F., Baker, H.M., Norris, G.E., Rice, D.W. and Baker, E.N. (1989) *J. Mol. Biol.* 209, 711.

3. Bailey, S., Evans, R., Garratt, R.C., Gorinsky, B., Hasnain, S., Horsburgh, C., Jhoti, H., Lindley, P.F., Mydin, A., Sarra, R.and Watson, J.L. (1988) *Biochemistry* 27, 5804.
4. Lindley, P.F., Bajaj, M., Evans, R.W., Garratt, R.C., Hasnain, S.S., Jhoti, H., Kuser, P., Neu, M.; Patel, K., Sarra, R., Strange, R. and Walton, A. (1993) *Acta Cryst.*, D49, 292.
5. Harris, W.R. and Madsen, L.J. (1988) *Biochemistry* 27, 284.
6. Harris, W.R., Sheldon, J. (1990) *Inorg. Chem.* 29, 119.
7. Grossmann, J.G., Neu, M., Pantos, E., Schwab, F.J., Evans, R.W., Townes-Andrews, E., Lindley, P.F., Appel, H., Thies, W-G. and Hasnain, S.S. (1992) *J. Mol. Biol.* 225, 811.
8. Grossmann, J.G., Neu, M., Evans, R.W., Lindley, P.F., Appel, H. and Hasnain, S.S. (1993) *J. Mol. Biol.* 229, 585.
9. Cowart, R.E., Kojima, N. and Bates, G.W. (1982) *J. Biol. Chem.* 257, 7560.
10. Bali, P.K., Zak, O. and Aisen, P. (1991) *Biochemistry* 30, 324.
11. Kretchmar Nguyen, S.A., Craig, A. and Raymond, K.N. (1993) *J. Am. Chem. Soc.* 115, 6758.
12. Dobbin, P.S. and Hider, R.C. (1990) *Chem. Brit.* 565.
13. Jones, M.M. (1983) *Met. Ions Biol. Sys.* 16, 47.
14. Funk, W.D., MacGillivray, R.T.A., Mason, A.B., Brown, S.A. and Woodworth, R.C. (1990) *Biochemistry* 29, 1654.
15. Woodworth, R.C., Mason, A.B., Funk, W.D. and MacGillivray, R.T.A. (1991) *Biochemistry* 30, 10824.
16. Crichton, R.R., Ward, R.J. (1992) *Biochemistry* 31, 11255.
17. Trowbridge, J.S. in *Monoclonal Antibody Therapy. Prog. Allergy.* Waldmann, H. ed., Basel, Karger 1988, 45, 121-146.
18. Olivieri, N.F., Koren, G., Louis, P.S., Freedman, M.H., McClelland, R.A. and Templeton, D.M. (1990) *Semin. Hematol.* 27, 101.
19. Kontoghiorghes, G.J., Aldouri, M.A. and Hoffbrand, A.V. (1987) *Br. Med. J.* 295, 1509.
20. Hayes, R.L. and Hubner, K.F. (1983) *Met. Ion. Biol. Sys.* 16, 279.
21. Lushbaugh, C.C. and Washburn, L.C. (1979) *Health Phys.* 36, 472.
22. Weitl, F.L., Harris, W.R. and Raymond, K.N. (1979) *J. Med. Chem.* 22, 1281.
23 Aramini, J.M. and Vogel, N.J. (1993) *J. Am. Chem. Soc.* 115, 245-252.
24 Kubal, G., Sadler, P.J. and Evans, R.W. (1992) *J. Am. Chem. Soc.* 114, 1117-1118.
25 Farrar, G., Altmann, P., Welch, S., Wychrij, O., Ghose, B., Lejeune, J., Corbett, J., Prasher, V., and Blair, J.A. (1990) *Lancet* 335, 747-750.
26 Barabas, K., Sizensky, J.A. and Faulk, W.P. (1992) *J. Biol. Chem.* 267, 9437.
27 Yeh, C.J.G., Taylor, C.G. and Faulk, W.P. (1984) *Clin. Immunol. Immunopathol.* 32, 1.
28 Wellhoner, H.H., Neville, D.M., Srininvasachar, K. and Erdmann, G. (1991) *J. Biol. Chem.* 266, 4309.
29 Kratz, F., Hartmann, M., Bertini, I., Keppler, B. and Messori, L., *J. Biol. Chem.* , in press.
30. Cazzola, M., Bergamaschi, G., Dezza, L. and Arosio, P. (1990) *Blood* 75, 1903.
31. Chitambar, C.R., Zivkovic, Z. (1987) *Cancer Res.* 47, 3929.
32. Chitambar, C.R. and Seligman, P.A. (1986) *J. Clin. Invest.* 78, 1538.
33. Chitambar, C.R., Matthaeus, W.G., Antholine, W.E., Graff, K. and O'Brien, W.J. (1988) *Blood* 72, 1930.

**Received: September 2, 1993**

